# Emerging brain organoids: 3D models to decipher, identify and revolutionize brain

**DOI:** 10.1016/j.bioactmat.2025.01.025

**Published:** 2025-02-12

**Authors:** Yuli Zhao, Ting Wang, Jiajun Liu, Ze Wang, Yuan Lu

**Affiliations:** a College of Life Sciences, Shenyang Normal University, Shenyang, 110034, Liaoning, China; b Department of Chemical Engineering, Tsinghua University, Beijing, 100084, China; c Key Laboratory of Industrial Biocatalysis, Ministry of Education, Tsinghua University, Beijing, 100084, China; d Tianjin Industrial Microbiology Key Laboratory, College of Biotechnology, Tianjin University of Science and Technology, Tianjin, 300457, China

**Keywords:** Brain organoids, Organoid culture, Brain microenvironment, Brain disease modeling, Organoid intelligence

## Abstract

Brain organoids are an emerging *in vitro* 3D brain model that is integrated from pluripotent stem cells. This model mimics the human brain’s developmental process and disease-related phenotypes to a certain extent while advancing the development of human brain-based biological intelligence. However, many limitations of brain organoid culture (e.g., lacking a functional vascular system, etc.) prevent *in vitro*-cultured organoids from truly replicating the human brain in terms of cell type and structure. To improve brain organoids' scalability, efficiency, and stability, this paper discusses important contributions of material biology and microprocessing technology in solving the related limitations of brain organoids and applying the latest imaging technology to make real-time imaging of brain organoids possible. In addition, the related applications of brain organoids, especially the development of organoid intelligence combined with artificial intelligence, are analyzed, which will help accelerate the rational design and guidance of brain organoids.

## Highlights

• Brain organoids are highly promising 3D models for *in vitro* studies.
• They are used in personalized medicine, intelligent learning and cutting-edge fields.
• The brain organoid culture technology and detection means are developing.
• The challenges include brain organoid flux, repeatability and long-term stability.
• Brain organoids' bionic function and diverse applications are expanding.

## Graphical abstract

**Figure:**
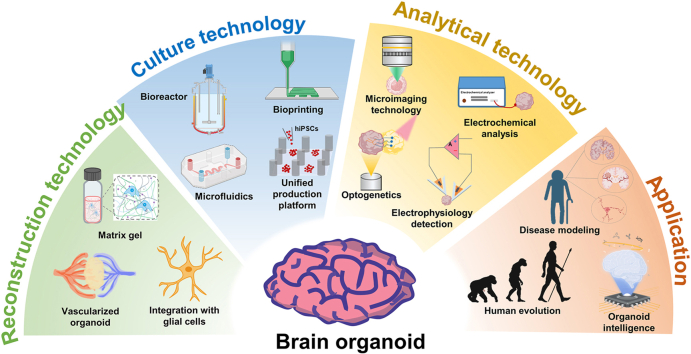

**Table:** Abbreviation index

Abbreviation	Full name
2D	two-dimension
3D	three-dimension
4D	four-dimension
ALS	amyotrophic lateral sclerosis
BBB	blood brain barrier
B-ECM	brain extracellular matrix
ChR2	channel retinoid-2
CNS	central nervous system
DA	dopamine
EB	embryoid body
ESCs	embryonic stem cells
fMOST	fluorescence micro-optical tomography
GFR	growth factor reduced
GLICO	cerebral organoid glioma
GSCs	glioma stem cells
hCOs	human cortical organoids
Hep-HA	heparin plus hyaluronic acid
hfMCOs	human fused MGE -cortical class organs
hiPSC	human induced pluripotent stem cell
hMGEOs	human medial ganglionic elevation-like organs
hMSCs	human mesenchymal stem cells
hOLS	human oligodendrocyte spheroids
hPSC	human pluripotent stem cells
hStrSs	human striatal spheroids
hThOs	thalamic organoids
IGF-1	insulin-like growth factor-1
iMGLs	human microglial-like cells
iPSCs	induced pluripotent stem cells
LSM	light-sheet microscopy
MEAs	multi-electrode arrays
MIP	molecularly imprinted polymers
MN	motor neuron
NPC	neural progenitor cells
OCT	optical coherence tomography
ONNs	organ-like neural networks
OPCs	oligodendrocyte progenitor cells
PD	Parkinson’s disease
PDGF-AA	platelet-derived growth factor-AA
PDMS	polydimethylsiloxane
RWV	rotating wall vessel
SBRs	stirred bioreactors
SN	sensory neurons
SOEA	soybean oil epoxide acrylate
TMD	transition metal disulfide compounds
VPA	valproic acid

## 1 Introduction

The brain is a central system with complex neural activities. The investigation of its developmental mechanisms has long been a major challenge for the scientific community. Because of the sophistication of the brain and the difficulty in sample acquisition, the research on the human brain is limited by ethics, which makes it difficult to convert basic research into clinical application. The brains of other primates and rodents are fundamentally different in size, shape, and cell composition from the human brain and do not possess the advanced cognitive functions of the human brain [[1], [2], [3]]. Therefore, phenomena and disease mechanisms elucidated by animal models cannot fully explain human disease. In addition, traditional 2D cell cultures cannot simulate tissues' spatial structure and complex interactions. 3D modeling is essential to overcome the limitations inherent in traditional research techniques for simulating brain characteristics. The emergence of organoids offers great potential for developing 3D brain organoids *in vitro*. Under proper culture conditions, this model can reconstruct the developmental and neurogenesis of the brain [4].

Brain organoids are 3D cell aggregates cultured *in vitro* that mimic certain aspects of brain organization and development. Compared to 2D cultures, cellular interactions are more complex, involving intercellular contacts, signaling, and extracellular matrices, all of which better mimic cellular networks, spatial structures, gene expression, and related functions in the brain. However, existing culture techniques are limited. Brain organoids still need to be improved in terms of survival time, size, and complexity, and are not yet able to mimic the characteristics of a fully real brain [5,6].

The development of brain organoids follows a “default program” that is driven by intracellular gene expression and tissue autonomy. Pluripotent stem cells, induced by specific growth factors, first form neuroepithelial cells, which then further differentiate into neural progenitor cells, proliferating and progressively differentiating into different types of nerve cells and neurons [[7], [8], [9], [10]]. Neurons then spontaneously arise and begin to form the initial neural network, a process that is self-organizing. Once the default program has established the basic brain structure, further regionalization and the formation of specific brain regions require the intervention of external factors. Through this combination of intrinsic programs and external factors, brain organoids not only mimic the proliferation and differentiation of neural cells during embryonic brain development [11], but also provide a basis for exploring cellular interactions in spatial structures.

Currently, the methods mainly used for brain organoid construction include two main categories. The first method is self-organizing. It relies solely on the spontaneous morphogenesis and intrinsic differentiation capacity of hPSC aggregates. This method usually generates brain organoids containing multiple brain regions, such as forebrain, midbrain, hindbrain, retina, choroid plexus, etc. [12]. These brain organoids can often mimic the interactions and developmental processes of multiple brain regions. However, it should be worth noting that, as the method is based on the self-modeling of developmental processes, there is high variability in the spatial properties of neural ectodermal regions originating from brain organoids [13]. The second method is directed differentiation, which is based on the principle of regulation of brain development, and precisely controls key points during the culture process to induce the formation of specific brain regions by supplementing exogenous morphogenetic and neurotrophic factors. This method reduces the variation in the same batch through the introduction of exogenous signaling molecules, which allows the generated organoids to more closely resemble the target brain region in terms of structure and properties. As a result, the directed differentiation process exhibits higher specificity and consistency compared to self-organization methods. However, the directed differentiation technique also faces some challenges, including the complexity of the manipulation steps, higher costs, and potential functional limitations.

The development of brain organoid technology has driven its application in scientific research and disease modeling. It provides physiologically relevant models for exploring human brain development and reveals developmental trajectories through single-cell technology [14]. Researchers understand neural networks by observing their neuronal activity, and patient-derived brain organoids show potential for disease diagnosis and drug screening, advancing personalized medicine [9,15]. In addition, brain organoids are used to study brain injury and neural regeneration, with midbrain organoids of dopaminergic neurons offering new hope for Parkinson’s disease treatment.

Although brain organoids show good promise for various applications, they still have many unresolved issues, including the absence of a vascular system, which may hinder their accuracy in specific applications. Since Lancaster’s use of whole-brain organoids in 2013 [9], brain organoid culture techniques have continued to improve and refine. However, current culture-generated brain organoids still lack some specific cells required for neurogenesis. Therefore, refining the cell types of brain organoids and integrating glial cells to improve their functionality remain key issues. In addition, the culture is usually limited by oxygen and nutrients, and the size of brain organoids is usually around 3–4 mm, which greatly limits the maturation of organoids and the accurate realization of related physiological functions. The realization of an organoid functional vascular network that can accurately reproduce the physiological functions of the human brain will provide a solid foundation for its widespread application.

**Fig. 1:**
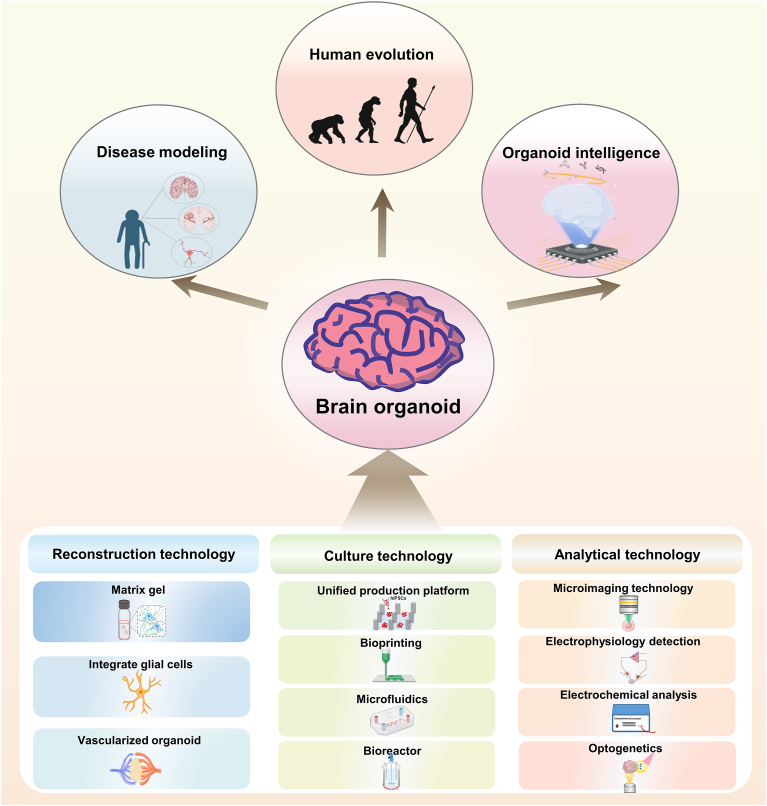
The main contents of emerging brain organoids. From *in vitro* complete reconstruction technology to cutting-edge culture technology (high-throughput platform, bioreactor, microfluidic, printing technology) and analytical detection technology (morphological to physiological and biochemical analysis), brain organoids have been widely used in the field of disease modeling, human evolution, and emerging organoid intelligence. Created with BioRender.com.

Starting from the necessity of bionic function enhancement of brain organoids, this paper reviews the application of the latest culture techniques and assays in improving their performance, and discusses the development of brain organoids in the new fields of disease modeling and organoid intelligence. Solutions to its limitations are also proposed. In addition, this paper provides an innovative overview of the latest culture techniques and trends in brain organoids, offering new perspectives for enhancing their bionic functions. For the first time, cutting-edge applications in the field of organoid intelligence are discussed in a brain organoid review, expanding its potential in learning cognition and intelligent computation. By identifying the limitations of brain organoids and proposing solutions, new ideas are contributed to the further development of the brain organoid research field. The main content of this paper is summarized in the figure shown below (Fig. 1).

## 2 Development of brain organoids

Organoid technology is a new type of tissue and organoid culture technology *in vitro*, which introduces the self-assembly characteristics of stem cells into 3D cell culture under the background of deepening research on mammalian development, tissue homeostasis, extracellular matrix, and increasingly rich experience in stem cell culture. In 2009, the Hans Clevers team successfully cultured adult stem cells *in vitro* into the crypt and villus structure of the small intestine for the first time [16], officially opening this field. In 2013, stem cells were successfully used to construct liver, kidney [17], and brain organoids [18,19], which once again increased the attention in this field. In just a few years, multiple types of organs such as lungs, stomach, retina [20], fallopian tubes, blood vessels, pancreas, skin, and heart were constructed. With the rapid development of technology, organoids have gradually shown their development potential as a new biological model in various fields.

**Fig. 2:**
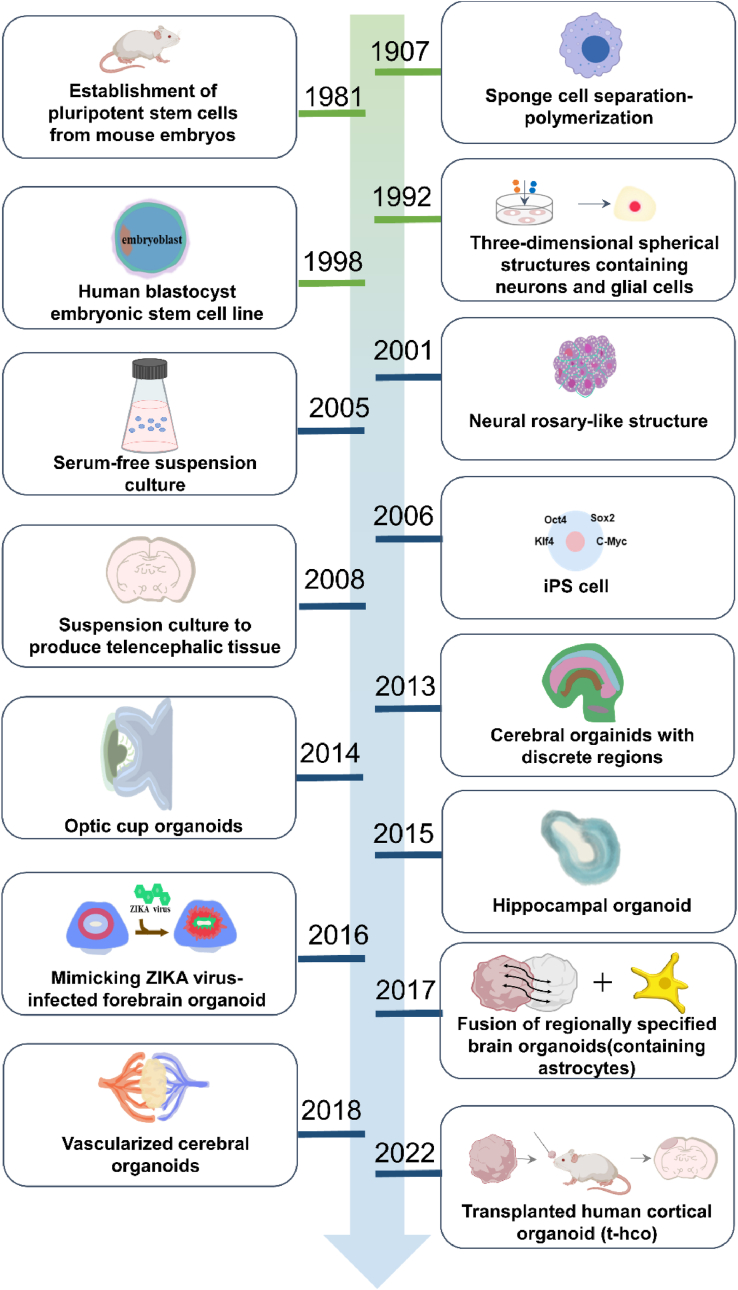
Historical processes and landmarks in the development of brain organoids. Wilson’s discovery that sponge cells have the ability to self-organize laid the foundation for organoid research [21]. With the advent and development of stem cell technology, Zhang et al. demonstrated that embryonic stem cells can produce neural rosette structures, an important intermediate in brain organoids [24]. To generate greater neural differentiation, suspension cultures, and rotating bioreactors were used to culture neural structures [25], and finally, in 2013, Lancaster et al. cultured a multilocular brain organoid, marking the successful establishment of a 3D brain organoid culture system [19]. Since then brain organoids have gradually converged towards the development of bionic brain organoids with cerebral organoids [12,[26], [27], [28]] and vascularized brain organoids [[29], [30], [31], [32]].

Advances in organoid technology drive brain organoid research. The development process of brain organoids is summarized (Fig. 2), which was based on Wilson’s first discovery of the self-organizing ability of sponge cells [21]. Based on this characteristic, brain organoids ushered in two milestone breakthroughs in the field of neurodevelopmental science after Martin et al. isolated pluripotent stem cells from mouse embryos [22]. First, Takahashi and Yamanaka discovered reprogramming factors that successfully induced pluripotency in various somatic cells [23]. Second, Zhang et al. demonstrated that embryonic stem cells could generate neural rosettes [24]. This neural rosette structure is an important intermediate in generating brain organoids. In order to generate greater neural differentiation, Yoshiki Sasai’s team pioneered the establishment of a serum-free suspension culture (SFEB) method, which successfully differentiated mouse embryonic stem cells (mESCs) generated prosopoietic embryoid bodies into telencephalic tissues by the addition of neural differentiation-inducing factors [25]. Subsequently, Lancaster et al. used a rotating bioreactor to increase the exchange of gases and nutrients, embedded the induced differentiated embryoid bodies in matrix gel, and finally differentiated them to form a brain organoid containing multiple brain regions by shaking and culturing them in a neural differentiation medium, marking the successful establishment of the 3D brain organoid culture system [19].

The initial establishment of *in vitro* models has driven the direction of brain organoid refinement. Based on Lancaster’s whole-brain organoid culture method, Qian et al. developed an improved brain region-specific organoid platform with higher reproducibility, simplicity, and economy [26]. The combination of micro-rotating bioreactors and the application of inducible factors made it possible to derive region-specific organoids from human iPSCs, including forebrain, midbrain [27] and hypothalamic-specific organoids [12]. Region-specific organoids drive exploration of unique features of different brain regions, such as forebrain organoids, that can be used to study the intrinsic program of human neocorticogenesis [28].

Since the introduction of different culture strategies, brain organoids are moving toward more functionality. To generate functional vascular networks, Mansour et al. achieved functional synaptic connectivity in rats through a xenograft technique [29]. However, functional vascular integration has not yet been achieved using co-culture techniques [30,31]. In addition, the human brain is typically characterized by a mature and complex neural network. Pasca’s team constructed transplanted human cortical brain organoids that mature normally in rats and integrate into neural circuits, providing a new platform for neurodevelopmental and disease research [32]. From self-tissues to stem cells and then to brain organoids, the continuous development of *in vitro* culture technology has made it possible to cultivate more biochemical *in vitro* models.

## 3 Reconstruction techniques for improving the functionality of brain organoids

**Fig. 3:**
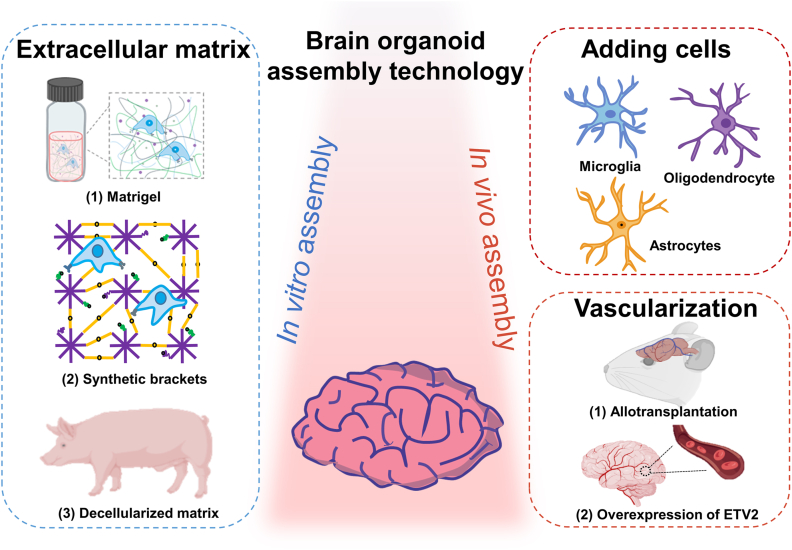
Reconstruction technology of brain organoids. *In vitro* culture of brain organoids requires additional *in vitro* matrix glue (different sources) and *in vivo* addition of glial cells (oligodendrocytes, astrocytes, and microglia) as well as vasculature (overexpression of endothelial cytokines or xenotransplantation). Created with BioRender.com.

Most programs produce brain organoids that are structurally limited in their functioning. Brain organoids usually lack non-epidermal cell types such as microglia [33]and the vascular system [31,34]. At the same time, cell culture methods ignore the important cell-cell and cell-matrix interactions regulated by the extracellular microenvironment. Therefore, this section summarizes reconstruction techniques for improving brain organoid function, including integrating non-outer epidermal cells, constructing vascular systems, and improving the extracellular matrix (Fig. 3).

### 3.1 Extracellular matrix scaffolds providing support and signaling for organoid development

**Fig. 4:**
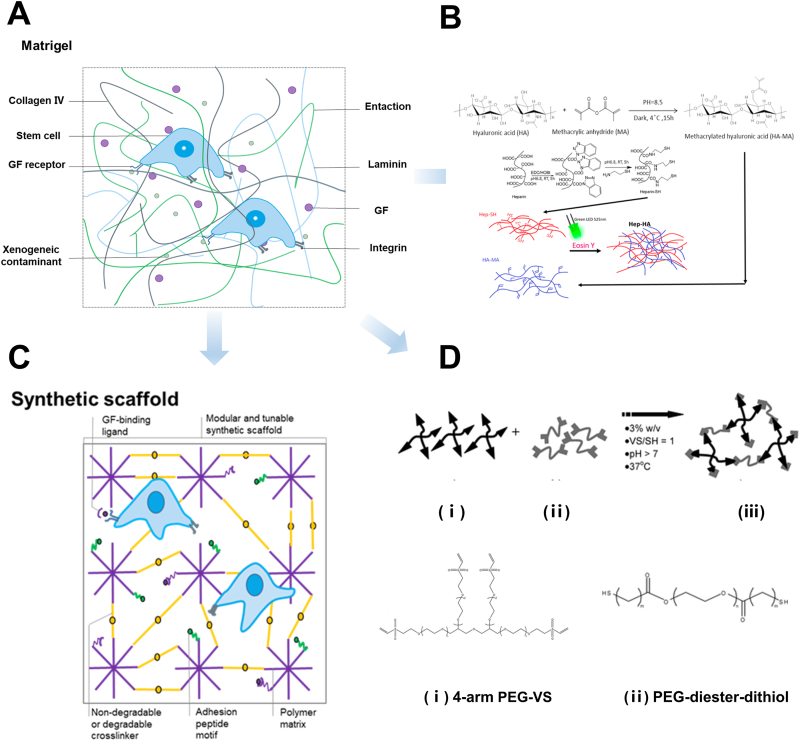
Natural and synthetic hydrogel scaffolds for brain organoid culture (Arrows indicate gradual expansion from natural hydrogels to different types of hydrogels). (A) Matrigel, the most commonly used natural hydrogel, provides support and critical biosignals for organoid culture [36]. (B) Different neural patterns of pluripotent stem cells induced by Hep-HA hydrogel formed from hyaluronic acid and heparin. Reprinted with permission. Copyright 2018, ACS Biomater Sci Eng [57]. (C) Synthetic scaffolds possess structures that define their chemical properties and enable tuning of mechanical, biological, and physical parameters, guiding organoid formation [36]. (D) Diagrammatic illustration of the cross-linking process for PEG hydrogels. A four-arm PEG-VS precursor polymer solution (i) was mixed with PEG-diester-dithiol crosslinker (ii) to obtain a 3D hydrogel within physiological settings (iii). Reprinted with permission. Copyright 2010, ACS [60].

The extracellular matrix (ECM) of the brain is a complex network structure that fills the space between neuronal cells and non-neuronal cells and consists mainly of structural proteins, proteoglycans and adhesive proteins. These components provide structural support and direct cellular activity, and are essential for brain development. The ECM commonly used in current *in vitro* protocols for generating organoids [35,36], Matrigel, a common extracellular matrix currently used to generate organoids *in vitro*, is a soluble basement membrane matrix derived from mouse tumors that supports the growth and differentiation of certain types of cells (Fig. 4A). However, it may not fully meet all the needs of brain development [[37], [38], [39]]. To mimic the real brain microenvironment, a variety of natural and synthetic organoid scaffolds derived from ECM have been created. Table 1 summarizes the different extracellular matrix scaffolds. These synthetic scaffolds lay the foundation for the production of highly biochemical brain organoid models.

**Table 1:** Comparison of different extracellular matrix scaffolds.

Material classification	Materials	Characteristics	Shortcomings	Reference
Natural ECM	Matrigel	The ability to mimic the *in vivo* mechanical and chemical characteristics of the ECM, while also offering essential signaling cues through various basement membrane ligands.	The ingredients are complex and difficult to control.	[35,36,38,39]
	Decellularized ECM	Has key components of ECM and good biocompatibility.	High heterogeneity and difficulty in changing the physical properties of the culture system without changing the chemical concentration.	[40,41]
	HA	Has a variety of biologically active molecules with ligands that bind to neural stem or progenitor cells and can improve neural progenitor cell graft survival.	High heterogeneity and difficulty in changing the physical properties of the culture system without changing the chemical concentration	[[42], [43], [44], [45], [46]]
Synthetic hydrogel	Polyacrylamide (PAM) and polyethylene glycol (PEG)	The structure is tunable and chemically defined to facilitate organoid formation across different parameters.	/	[[47], [48], [49]]
	Self-assembling peptides	Has a chemically defined structure that can be adapted to biological, mechanical and physical parameters and can provide certain specific functions.	/	[47,50]

To understand the composition and structure of the natural ECM during brain development, the researchers attempted to use human and animal acellular ECM to culture brain organoids. Decellularized ECM effectively retains key ECM components, including elastin, fibronectin, collagen type I, and laminin, and exhibits excellent biocompatibility [40]. This provides an optimal tissue environment and tissue-specific ECM signals for developmental organoids, facilitating stem cell differentiation and reprogramming. Robin et al. processed decellularized adult porcine brain extracellular matrix (ECM) into hydrogel scaffolds for brain organoids culture [41]. The study showed that this B - ECM hydrogel had similar effects to Matrigel in supporting brain organoids in culture, mimicking the brain microenvironment, and promoting neurogenesis and maturation.

In addition to decellularized ECM, several laboratories have also employed biomacromolecular polysaccharides, including hyaluronic acid and its mixtures with chitosan, to support the cultivation of brain organoids [46,[51], [52], [53], [54], [55]]. Hyaluronic acid (HA) is an important component of the ECM [42]. Bioactive molecules and ligands bind to neural stem or progenitor cells, thus contributing to nerve cell survival [[43], [44], [45]]. It is because of its well-defined composition and function that hyaluronic acid is widely used for neural cultures. Lindborg et al. used hyaluronic acid and chitosan to prepare electrostatically cross-linked hydrogels that promoted the formation of cortical-like structures in brain organoids without the need for additional neural-inducing components [56]. Bejoy et al. found that heparin-conjugated hyaluronic acid hydrogels better induced ectodermal differentiation of hiPSCs (Fig. 4B), and that the hardness of the hydrogel affected the direction of stem cell differentiation, with low modulus promoting forebrain fates and high modulus promoting hindbrain fates [57].

The heterogeneity and difficulty of controlling the physical properties of biologically derived decellularized matrices and hyaluronic acid limit the alteration of their mechanical properties. Therefore, the researchers used synthetic hydrogels to culture brain organoids for precise control. Synthetic hydrogels have chemically defined structures that can be adjusted to meet specific biological, mechanical, and physical criteria, allowing for precise control over organoid formation [58]. These synthetic hydrogels usually contain cell adhesion structural domains or protein hydrolysis degradation sites (Fig. 4C). Most synthetic scaffolds currently consist of polyacrylamide (PAM) and polyethylene glycol (PEG). Of these, it is widely used because of its hydrophilic, biologically inert, and highly resistant to chemical modification (Fig. 4D) [48,49,59,60]. Schwartz and colleagues used PEG-based gels to generate neural organoid tissue [61].

Synthetic hydrogels, in addition to utilizing the two polymers mentioned above, can also be used to guide cell differentiation by adding peptides to provide specific biological functions. For example, self-assembling peptide (SAP) nanofiber hydrogels incorporate peptide sequences from the brain’s extracellular matrix (ECM), which enables the inhibition of neuronal apoptosis and promotes the differentiation of stem cells [50]. Pugliese, Marchini et al. developed the HYDROSAP system, where multifunctional and branched SAPs form hydrogels with a controlled elastic modulus [47]. The authors used the system to culture human fetal neural stem cells and successfully differentiated them into neural cell types, showing great potential for development in neural regeneration and tissue engineering. Different extracellular matrix scaffolds provide the necessary support and signaling for the development of brain organoids through their respective unique structures and compositions. They are an integral part of the enhancement of bionic functions of brain organoids. However, Matrigel, decellularized matrix of biological origin, and hyaluronic acid have their own defects. In the future, synthetic hydrogels and self-assembled peptide hydrogels may have greater potential for development in brain organoid culture.

### 3.2 Addition of glial cell populations that promote neurogenesis

Gliogenesis accompanies neurogenesis during brain development [62]. Glial cells account for roughly half of the total cells in the brain and include astrocytes, oligodendrocytes, and microglia. They promote functional synapse formation, support the maintenance of neuronal signaling capacity, and play a key role in CNS and disease progression. Existing studies have shown that mature oligodendrocytes can be generated *in vitro* [[63], [64], [65]] and that brain organoids containing microglia-like cells can be generated by co-culture. Due to the special origin of microglia, this subsection briefly describes the current special induction process of differentiation to form microglia and several strategies to generate microglia-containing brain organoids.

Microglia are the major neuroimmune cells in the brain and are involved in several neurological processes. They originate in the mesoderm and differentiate from red myeloid progenitor cells in the embryonic yolk sac [66,67], migrating to the brain to develop into microglia. This subsection focuses on differentiation strategies from induced microglia-like cells [68], involving timed exposure to growth factors or small molecule chemicals (e.g., CSF1 or IL34). To shorten the differentiation cycle, the researchers introduced SPI1 and CEBPA/B into human pluripotent stem cells, skipping the progenitor cell stage. To ensure microglia stability and function, maintenance factors such as CX3CL1, CD200, and TGF-β [69], which play key roles for microglia in synaptic pruning and neural network health, were also added [70].

Several strategies for generating three-dimensional brain organoids containing microglia are briefly discussed here. That is, exogenous microglia or microglia precursor cells (MPCs) are co-cultured with brain organoids to generate microglia-like cell-containing brain organoids [7,67,68,[71], [72], [73]]. However, given that neural precursor cells (NPCs) have the ability to self-assemble into 3D brain organoids [74], co-culture of human MPCs with NPCs can generate brain organoids containing controlled numbers of iMGs. Region-specific integration of microglia was achieved by co-culturing human pluripotent stem cell-derived primitive neural precursor cells (pNPCs) and primitive macrophage precursor cells (PMPs) [71]. The model was able to regulate the number of microglia and was able to demonstrate their function in phagocytosis and synaptic pruning. Furthermore, by adjusting the concentration of heparin and delaying the embedding of the matrix gel in the organoids, Ormel et al. found that brain organoids cultured in an unguided protocol without the use of dual SMAD signaling inhibition could spontaneously form microglia [75]. Although existing strategies are capable of generating microglia in organoids, the extent to which these induced generated cells are able to mimic cells *in vivo* and elicit a consistent response has not been fully determined. Differences in maturity and responsiveness to immune stimuli of microglia used in different studies may also contribute to the variability of findings.

### 3.3 Naturally perfusable brain organoids

Stem cell-derived brain organoids (COs) lack functional vascular systems; yet these functional vascular systems are critical in neuromodulation and brain development, which severely limits the size and maturity of brain organoids [[76], [77], [78], [79]]. Functional neurovascular networks are mainly involved in the proliferation and differentiation process of neural progenitor cell populations through dynamic paracrine crosstalk exchanges, and the construction of functional neurovascular networks is crucial for building more accurate models of the human brain. People employed various approaches to construct functional vascularized networks using different approaches, such as ectopic expression of human ETS variant 2 (ETV2) [31], organoid endothelialization [80,81], vascular organoid fusion [82,83], xenografts [84], and emerging microarray technologies [85,86], also with varying degrees of achievement.

**Fig. 5:**
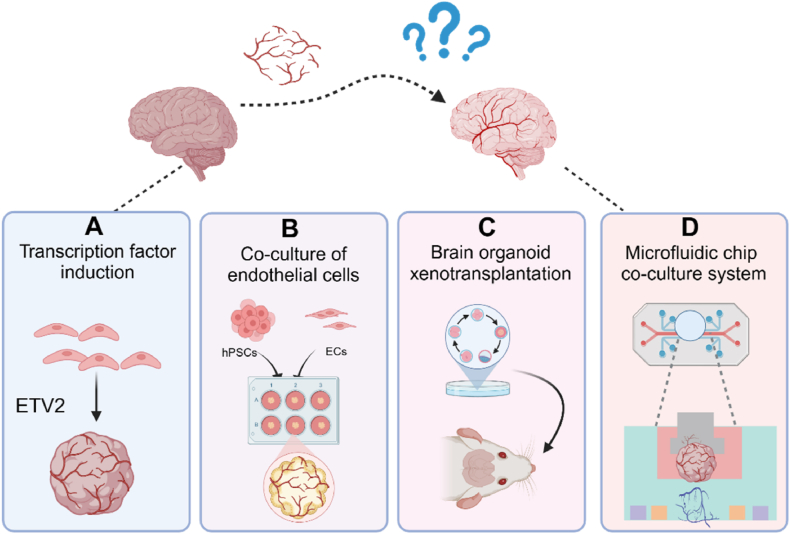
Methods for constructing vascularized brain organoids. (A) Overexpression of the ETV2 gene generates vascularized brain organoids [30,31]. (B) Brain organoids were co-cultured with ECs to generate vascular system [30]. (C) Brain organoids were able to fuse with the host vascular system after xenografting [88]. (D) Schematic of brain organoids cultured using microfluidic chip technology [95]. Created with BioRender.com.

Functional vascular networks have not been achieved by the overexpression of the ETV2 gene or by co-culturing induced hPSCs with endothelial cells (HUVEC) [87]. Although overexpression of ETV2 in hCOs contributes to the formation of a vascular-like network (Fig. 5A), it lacks functionality *in vitro* and requires transplantation to form a functional vascular network [31]. In addition, protocols to construct functional vascularized brain organoids by co-culturing organoids with endothelial cells (ECs) or vascularized organoids [82] have shown to produce brain organoids with complex tubular vasculature and functional neurovascular units (Fig. 5B) [30], but have also failed to result in the formation of a desirable functional vascular network.

To solve this problem, the simplest research idea is to transplant brain organoids into rodent brains by means of xenotransplantation, using the host’s vascular network to facilitate the maturation and refinement of the organoids (Fig. 5C). Thus, it helps to overcome the limitations of long-term culture [88]. Mansour and colleagues implanted brain organoids into rat brains. The host vasculature system was utilized to promote the growth and maturation of the brain organoids and to achieve synaptic connections within the host brain [29]. Although xenotransplantation can provide a more realistic physiological environment for brain organoids to promote the maturation and bionic function of brain organoids, it can also provide a new way for the research and treatment of neurodegenerative diseases and brain injuries [89]. However, since transplanted brain organoids use the host’s vascular network to promote their own maturation and refinement, the problem of xenocontamination may exist.

To solve the above limitations, the brain organoid chip system, which combines brain organoids with microfluidic technology, provides a new culture method to improve the perfusability of brain organoids [90,91]. Microfluidic technology can simulate the physiological environment of human brain development by precisely controlling various parameters of the culture system [92]. In addition, by engineering the vascular system to perfuse tissue structures, vascular microenvironmental cues can be precisely controlled *in vitro*, resulting in the construction of perfusable microvessels that are functionally and size-wise similar to human microvessels [85,93,94]. Salmon et al. used a custom-designed 3D-printed microfluidic chip to form integrated neurovascular organoids (Fig. 5D) [95]. This system can be used to study the interaction of brain organoids with the vascular system. However, the formation of a perfusable system opens up the possibility of delivering substances such as immune cells or drugs to brain organoids to probe brain development and pathogenesis. Using the brain organoid microarray system to simulate neurodevelopmental deficits under prenatal nicotine exposure, it was found that nicotine exposure leads to abnormal neuronal differentiation and migration, demonstrating that nicotine exposure leads to impaired neurodevelopment in the fetal brain [96]. Overall, the brain organoid microarray system not only serves as a valuable model for investigating the impact of prenatal nicotine exposure on neurodevelopment, but also opens up new avenues for future brain disease research and drug testing.

## 4 Engineering technologies to improve the functionality of brain organoids

iPSCs-derived organoids are capable of encompassing the earliest stages of brain development and can serve as good brain organoid models. However, organoid technology still faces multiple challenges, such as heterogeneity and limitations of cell differentiation during culture and long-term culture, making it difficult to form homogeneous brain organoids for subsequent research applications. To improve the culture techniques and reduce the variability caused by human manipulation, researchers have used engineering techniques such as bioprinting, microfluidics, and bioreactors to improve organoid culture systems and environments, which can help improve brain organoid functionality. Table 2 summarizes the advantages, disadvantages, and application domains of various engineering methods aimed at enhancing brain organoid functionality are encapsulated.

**Table 2:** Comparison of engineering techniques to improve functionality.

Engineering technology	Equipment		Characteristics	Shortcomings	Applications	Reference
Production technology	Droplet method		Easy to operate and suitable for mass production.	There is no way to control the size and traits of embryoid bodies (Ebs).	Generate EBs.	[97,98]
	Static suspension method		Precise control of EBs formation and differentiation to produce uniform EBs.	Not suitable for mass production applications.	Generate uniform EBs.	[99]
	Bioreactor		Not only improves the circulation of nutrients and cellular waste but also prevents cell aggregation. Suitable for mass production.	Dependent on the cell’s own differentiation.	Generate uniform EBs.	[[100], [101], [102], [103]]
	Microfluidic chip-based droplet technology		Efficient mass production of EBs	Dependent on cell differentiation.	Generate uniform EBs.	[104]
Bioprinting	Inkjet-based bioprinting technology		Controlled printing. Not only does it preserve the properties and functions of the cells themselves but it also reveals functional stimulus-responsive neural networks.	/	Brain organoids	[105,106]
	Extrusion-based 3D bioprinting.		Micrometer resolution, constructs remain pluripotent.	/	Fabrication of highly coherent ovoid spheres and organizations with long structures	[105,107]
	4D printing technology		Over time, stimuli can be applied to the printed structure to make it resemble physiological changes that occur during development	/	Brain organoids	[108,109]
Engineering techniques to improve oxygen and nutrient supply	Microfluidics	Schematic diagram of the 3D incubation zone and channel microfluidic device.	Fluid flow enhances nutrient and oxygen exchange in channels for EBs in hydrogel.	/	Brain organoids	[93,104,[110], [111], [112], [113], [114], [115]]
		Schematic diagram of a microcolumn array microfluidic device.	Ability to generate brain organoids *in situ*, reducing the harvesting step for artificially transferred EBs.			
		Schematic of a microfluidic device at the air-liquid interface.	Has physical constraints, improving size repeatability			
	Bioreactor	Stirred bioreactors (SBRs)	This type of bioreactor improves organoid culture mainly through improved sensing and enhanced oxygenation	The system requires much media and culture space.	Brain organoids	[19,[116], [117], [118], [119], [120]]
		Rotating wall tube bioreactors (RWVs)	The reactor is gentle and has low shear mixing characteristics.	/		

### 4.1 Technology platform for large-scale harmonized production of embryoid bodies

Most protocols for generating organoids begin with hPSC aggregates, referred to as EBs. Generating high-throughput scalable embryoid bodies is a key step in organoid culture, as the growth status of embryoid bodies is closely tied to the developmental progression and functional capacity of the resulting organoid.

**Fig. 6:**
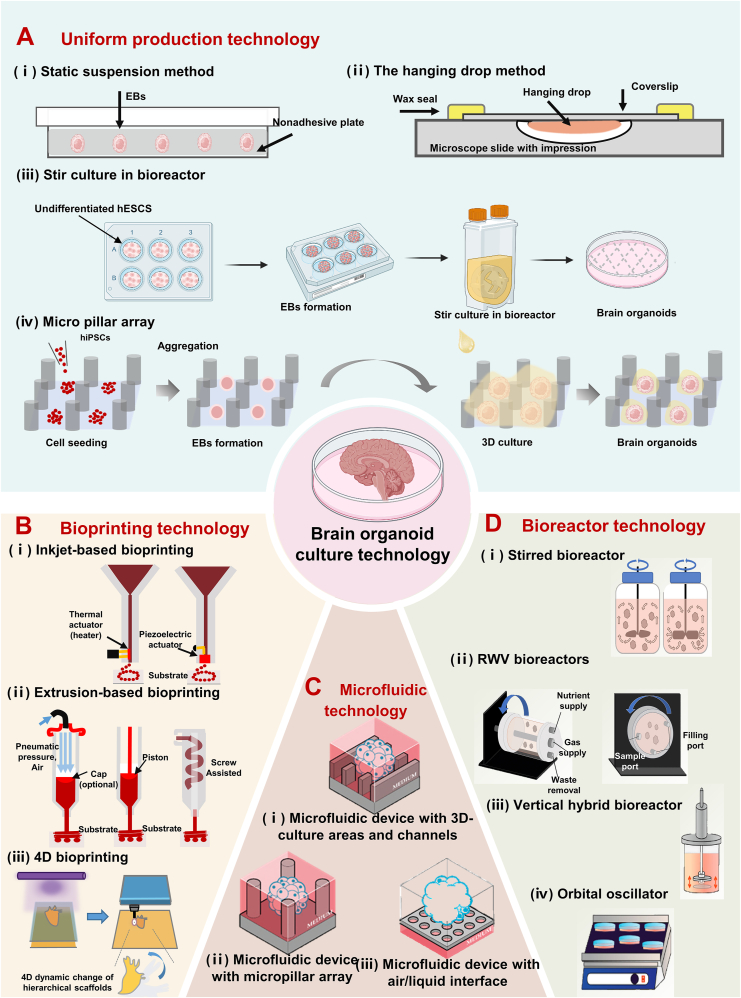
Schematic diagram of engineering techniques used to improve brain organoid culture. (a) Production of scalable and homogeneous EBs technology. ⅰ) Static suspension method. ⅱ) The hanging drop method. ⅲ) Stir culture in bioreactor. ⅳ) Micro pillar array. (b) Widely used bioprinting technology. ⅰ) Inkjet-based bioprinting technology. ⅱ) Extrusion-based 3D bioprinting. ⅲ) 4D printing technology: tandem lithography-stereolithography process for fabricating heart-shaped structures from a novel soybean oil epoxide acrylate. Reprinted with permission [108]. Copyright 2019, International Journal of Smart and Nano Materials. (c) Schematic of three microfluidics used for brain organoids culture. ⅰ) Schematic diagram of the 3D incubation zone and channel microfluidic device. ⅱ) Schematic diagram of a microcolumn array microfluidic device. ⅲ) Schematic of a microfluidic device at the air-liquid interface. Reprinted with permission [93]. Copyright 2022, Pharmaceutics. (d) Schematic diagram of a bioreactor commonly used for brain organ culture. (i) A stirred bioreactor with axial and radial impellers on the left and right. ii) Schematic diagram of two Rotating wall vessel bioreactors. iii) Schematic diagram of two types of stirred bioreactors. The arrows in the figure represent the direction of rotation. Reprinted with permission [121]. Copyright 2023, International Journal of Molecular Sciences. ⅲ) Vertical hybrid bioreactor. ⅳ) Orbital oscillator. Reprinted with permission [122]. Copyright 2022, American Chemical Society. Created with BioRender.com.

Widely used EB formation methods include suspension droplet and static suspension methods [123]. Dispersing the cells in suspension in a specific volume of Petri dish allows the process of EB formation and differentiation to be artificially controlled (Fig. 6A(ⅰ)) [97]. Since the blastomeres formed in each droplet are physically separated, individual blastomeres cannot aggregate, thus contributing to uniform cell growth and differentiation [98]. However, technical difficulties and limited space in scaling up droplet formation make this suspension droplet method unsuitable for large-scale applications. On the other hand, suspension culture is easy to operate, and the cells can spontaneously aggregate into spheres, suitable for large-scale production (Fig. 6A(ⅱ)). Therefore, it is widely used in the scale production of embryoid bodies [99]. Although this method can produce more embryoid bodies than the suspension droplet method, it tends to result in uneven aggregation of embryoid bodies because it relies on their spontaneous aggregation.

As technology advances, bioreactors, and microfluidics have emerged as effective solutions that bridge the gap between the control offered by suspension droplet methods and the ease of suspension culture techniques. Various bioreactors, including rotating vials, Slow Turning Lateral Vessels (STLV), and High Aspect Ratio Vessels (HARV), are increasingly employed for the large-scale production of EBs (Fig. 6A(ⅲ)) [100,101]. The agitation facilitated by bioreactors not only enhances the distribution of nutrients and the removal of cellular wastes but also allows for the precise control of cell aggregation [102,103]. Therefore, these bioreactors have proven effective in creating controlled environments that support the formation and differentiation of EBs. Similarly, microfluidic chip-based droplet technology has been shown to be useful for high-throughput preparation of EBs. In PDMS-based chips, microchannels are connected to open pores, and microfluidic control allows for the simultaneous formation of a large number of suspension droplets without the need to individually pipette the droplets and generate EBs expressing pluripotency markers in each droplet. Zhu et al. utilized a microcolumn structure to controllably fabricate EBs (Fig. 6A(ⅳ)) [104]. Cells aggregated between arrays of polydimethylsiloxane (PDMS) micropillars formed homogeneous, scalable EBs. A device platform capable of generating uniform and consistent EBs will enhance the scalability of brain organoid production, reduce variability, and markedly improve its function.

### 4.2 Bioprinting technology for reliable fabrication of biomimetic, intelligent living brain organoid models

Although high-throughput, fully automated screening platforms overcome the differences in human manipulation, the process of generating organoids still relies heavily on the differentiation ability of the cells themselves, an approach that sometimes fails to meet the research needs for high-precision control of the structure and function of specific tissues. Bioprinting technology, an advanced 3D tissue engineering method, is capable of accurately creating biological constructs with a hierarchical structure similar to that of natural tissues. The development of artificial tissues or organs tailored to the specific needs of patients is expected to address the needs of the healthcare field in tissue replacement and organ transplantation.

Commonly used bioprinting strategies include inkjet-based bioprinting and extrusion-based 3D bioprinting methods [105]. Inkjet bioprinting generally modifies commercial-grade inkjet printers to print living cells or biomolecules. This printing technique mixes cells and other biomaterials in a ‘bio-ink’ and then builds structures in a predetermined pattern to be ejected drop by drop from a nozzle onto a target surface (Fig. 6B(ⅰ)). Structures formed by this technique largely retain cellular properties and functions, such as neural phenotypes and electrophysiological properties, and may improve cell differentiation and survival as well as responsiveness to neural stimuli. Xu et al. demonstrated that neuronal cells with phenotypic and basic electrophysiological functions can be fabricated in a controlled manner by inkjet printing methods, using a commercial inkjet printer to directly eject rat primary embryonic hippocampal and cortical neurons into predefined monolayers of cellular structures [106]. Another commonly used printing technology is extrusion bioprinting, which involves extruding cell-laden bioinks through a nozzle with micrometer-scale resolution. This method is suitable for creating highly cohesive blastomeres and elongated structures (Fig. 6B(ⅱ)). Gu et al. constructed 3D constructs containing hiPSCs using extrusion-printed iPSC-containing polysaccharide-based bioinks [124]. The printed hiPSC constructs remain pluripotent, and when the 3D constructs are generated in a neural induction medium, the hiPSCs differentiate into various mature neurons and glial cells. Although 3D printing technology assumes a significant position in the precise construction of organoid structures, it still faces some limitations. For example, extrusion printing is relatively slow, which may affect large-scale applications. In addition, the resolution of extrusion printing is usually lower compared to inkjet printing technology, which may affect the fineness and complexity of its printing.

As an advanced fabrication technique, 4D printing has been used to construct brain organoids whose mechanical or physiological structures undergo multiple changes over time when these tissues are subjected to specific stimuli or trigger sources [108,109]. Thus, 4D printing technology can provide a scaffold for studying tissue or mechanical stress-induced changes in neuronal cell growth patterns and axonal tension, thereby elucidating the folding mechanisms of cortical tissues. Miao et al. developed multiresponsive 4D bioprinting based on light-cured molding using stereolithography with UV-crosslinked soybean oil epoxy acrylate (SOEA) (Fig. 6B(ⅲ)) [109]. This print not only has reversible shape change and shape memory properties, but is also capable of 4D transformation through thermo-mechanical programming. In addition, this 4D printing shows great potential for neuroregeneration applications, where human mesenchymal stem cells (hMSCs) were neurally differentiated by the addition of neural differentiation medium to the 4D printed architecture, and the results showed that the hMSCs were able to differentiate into neural cell types on the 4D-aligned graphene hybrid catheters. This breakthrough significantly advances the development of 4D printing technology in the field of neural tissue engineering.

### 4.3 Engineering techniques for precise control of nutrient and oxygen supply in culture processes

#### 4.3.1 Microfluidic technology

Conventional cell culture techniques have been well used in the past decades, but as organoids become larger, this culture method cannot meet the nutrient supply and gas exchange of their nuclei, which triggers the death of central progenitor cells. In order to address this challenge, microfluidic platforms have emerged as a leading and promising technological approach for the cultivation of organoids, which supports the continuous supply of medium through laminar flow, thereby reducing the “dead core” of brain organoids.

Microfluidic systems allow precise control of the microenvironment (e.g., flow rate, oxygen levels, pH) during organoid culture. They enhance oxygen supply and distribution, improve nutrient and waste exchange, and minimize shear stress on cells [93]. Lancaster et al. partially solved the challenge of angiogenesis in brain organoids by enhancing nutrient and oxygen uptake by brain organoid cells by culturing brain organoids in a microfluidic device [110]. In addition, another advantage of microfluidics is its design flexibility, allowing for the cultivation of more complex neural networks or the formation of multiple organ-on-chips by connecting multiple devices of different organoids [111,112]. An organoid chip in this context refers to a microfluidic cell culture device fabricated using computerized microchip fabrication technology, which consists of hollow microchannels that cultivate living cells and tissues in a physical environment associated with the organoid and are continuously injected with life-sustaining media.

Currently, three main microfluidic devices exist for organoid systems on a chip. The first 3D culture zone and channel microfluidic device, which consists of a 3D cell culture zone and a medium flow channel (Fig. 6C(ⅰ)), is used to improve cell viability, ameliorate cell necrosis in the central zone, and accelerate the maturation of organoids. Wang et al. utilized a microfluidic system to generate brain organoids from perfusable organoids [125]. Neuroectodermally differentiated EBs are encased in Matrigel and then placed in hydrogel channels that are perfused with medium through a central channel using a syringe pump. This directional fluid flow greatly facilitates the oxygen and nutrient exchange. Cho and his team created a microfluidic system that harnesses the hydrostatic pressure arising from the differing levels of medium in chambers connected through microchannels. This design employs a bi-directional rocking bar to generate cyclic fluid flow [126]. Brain organoids cultured with this device exhibited the development of cortical layers, increased volume, and significantly enhanced electrophysiological functions.

The second is a microcolumn array microfluidic device. The device consists of a number of microposts, between which cells can self-assemble into EBs, which in turn form organoids (Fig. 6C(ⅱ)). This microcolumn array device offers the advantage of generating brain organoids *in situ*, thus reducing the step of manually transferring EBs for harvesting. For example, Zhu et al. used *in situ* formation and differentiation of hiPSCs on an octagonal column microcolumn array to form functional human brain organoids [104], and Cui and colleagues examined the neurodevelopmental impacts of prenatal exposure to valproic acid (VPA) using their own brain organoids microarray platform combined with microcolumn arrays [113].

Additionally, a microfluidic device designed for operation at the gas-liquid interface is utilized. This microfluidic device promotes oxygenation of the culture medium, minimizing the formation of hypoxic cores within the organism. This device is also physically tuned to be able to control the size of the organisms to less than 2 mm to improve reproducibility (Fig. 6C(ⅲ)). Ao et al. utilized an integrated microfluidic platform for culturing brain organoids to investigate the effects of prenatal chemical exposure (PCE) on early brain development [115]. The platform integrates a perfusable culture chamber, a gas-liquid interface, and a simplified process to produce organoids in large quantities without complex assembly. This approach provides an efficient, scalable, and consistent method for culturing organoid tissues, with the potential to significantly enhance the functionality of brain organoids.

Microfluidics has shown great potential in optimizing brain organoid cultures. However, challenges remain in achieving high-throughput processing, high customization, ease of manufacturing, reproducibility of experiments, and design flexibility in 3D microfluidic cell culture systems. Looking ahead, continued advances in microfluidics are expected to further advance the field of brain organoid culture.

#### 4.3.2 Bioreactors

Similar to microfluidic devices, bioreactors enhance the uptake of nutrients and oxygen by brain organoids, offering a low-shear stress environment. This promotes the development of larger, more contiguous neural structures and supports the growth of substantial, intricate brain organoids. This subsection focuses on stirred bioreactors and rotating wall vessel bioreactors for the diffusion culture of brain organoids.

Stirred bioreactors (SBRs) usually consist of cylindrical culture vessels containing drivable impellers or agitators. This type of bioreactor improves organoid culture mainly through improved sensing and enhanced oxygenation (Fig. 6D(ⅰ)). Lancaster et al. showed that SBRs can generate larger and more continuous complex brain organoids from bioreactors than those grown under static conditions, improving oxygenation during organoid culture [9]. However, the system requires much media and culture space. To address this constraint, Qian et al. developed the miniaturized multi-well rotary bioreactor Spin Ω designed in conjunction with a 12-well plate, which improves oxygen and nutrient diffusion, thereby facilitating the formation of large continuous cortical structures [116]. Since each organoid is cultured in a separate well, the Spin Ω significantly boosts both the yield and consistency of organoid production.

Rotating wall tube bioreactors (RWVs) are also widely used. The bioreactor facilitates the rotation of organoids within a cylindrical vessel, delivering media in a controlled, low-shear environment while actively removing waste through perfusion (Fig. 6D(ⅱ)). DiStefano et al. demonstrated that the RWV bioreactor accelerates and improves organoid growth and differentiation [117]. Similar to the RWV bioreactor, The orbital oscillator generates a low-shear environment and supports separate Petri dishes for culturing limited numbers of organoids, empowering researchers to conduct parallel analyses of organoids under varying culture conditions (Fig. 6D(iv)) [118].

Typically, there are two types of RWVs used: a slow-turning lateral vessel (STLV) and a high-aspect-ratio vessel (HARV) (Fig. 6D(ⅲ)) [127]. Since the cell culture rotates with the entire vessel, the bioreactor provides a gentle and low-shear mixing environment [120]. Additionally, the rotational motion of the bioreactor is thought to promote faster growth and maturation of the organoids. DiStefano et al. cultured retinal organoids using an RWV bioreactor [127]. After initial 3D spheroid formation in static suspension, a subset of organoids was transferred to the RWV bioreactor, while a control group remained in static culture. The organoids in the RWV demonstrated rapid and accelerated growth and maturation, significantly outpacing those in static suspension. This enhanced growth and maturation are hypothesized to result from improved exchange of oxygen, nutrients, and waste. The application of engineering technologies to enhance organoid culture systems and environments can markedly improve the efficiency, stability, and functionality of organoid culture. These technologies not only provide brain organoids with conditions closer to the *in vivo* environment but also facilitate their maturation and functional development, thereby advancing their application in fields like drug discovery, disease modeling, and regenerative medicine.

## 5 Analytical techniques for examining the functionality of brain organoids

While the unobservability of the human brain has greatly hindered brain science research, advances in brain organoid technology have allowed to study their development *in vitro*. Frequent monitoring of organoids through imaging, as well as physiological and biochemical methods, to gather data on their morphological or developmental attributes is crucial in organoid studies. Especially for central nerves such as the brain, the ability to consistently detect electrophysiological signals within them may provide important insights into treating certain diseases. Table 3 summarizes the characteristics, current shortcomings, and application areas of different brain-based organoid analysis techniques.

**Table 3:** Comparison of different brain organoid analysis techniques.

Methods of analysis	Equipment	Characteristics	Application	Shortcomings	Reference
Orphological	Confocal microscopy	Fluorescent images showing expression of immune markers can be obtained, but penetration depth is limited (less than 100 μm).	Brain organoid section	Penetration depth is limited (less than 100 μm).	[128,129]
	Multiphoton microscopy	Imaging is faster, penetrates deeper, and reduces the risk of photobleaching and phototoxicity.	Brain organoid imaging	Imaging depth remains limited.	[130]
	Light microscopy	Higher contrast and resolution	Brain organoid imaging	Resolution, limited 3D imaging, photobleaching	[131,132]
	Real time imaging system	Formerly imaging, real-time tracking	Brain organoid imaging	/	[133]
	Optical coherence tomography	*In vivo* 3D imaging, long-term tracking, label-free	Brain organoid imaging	/	[134]
Electrophysiological	Membrane-clamp	Complex signal analysis captures individual neuron activity with high temporal accuracy	Electrical signals from a single neuron	Limited spatial resolution and analysis of network activities.	[135]
	Calcium imaging	Provides information about activity on a larger scale, but has low temporal resolution and is dependent on imaging equipment.	Electrical signals from hundreds of neurons.	Limited temporal resolution and difficulty in acquiring 3D imaging.	[136]
	3D microelectrode arrays	Three-dimensionality, high temporal resolution	3D electrical signals within organoids	Rigid metal electrodes may damage organoids and disrupt neural networks.	[137,138]
	3D flexible neural interface platform	The minimally invasive approach does not break the original neural network and has long-term stability.	3D electrical signals within organoids	/	[139]
	Stretchable mesh nanoelectronic components	Embedded nanodevices detect electrical signals throughout the development of organoids	3D electrical signals within organoids	/	[140]
Electrochemical	Electrochemical biosensors	Rapid and accurate detection of metabolic parameters and biomarker release.	For the detection of brain organoid metabolites and biomarkers.	/	[141]
	New electrochemical sensors	Non-invasive, label-free detection of biomarker release.	For the detection of brain organoid metabolites and biomarkers.	/	[142]
Optogenetic	Optogenetics	Regulation of cellular activity by light.	Explore the neurobiology of the brain and related diseases.	/	[[143], [144], [145]]

### 5.1 Morphological analysis methods

**Fig. 7:**
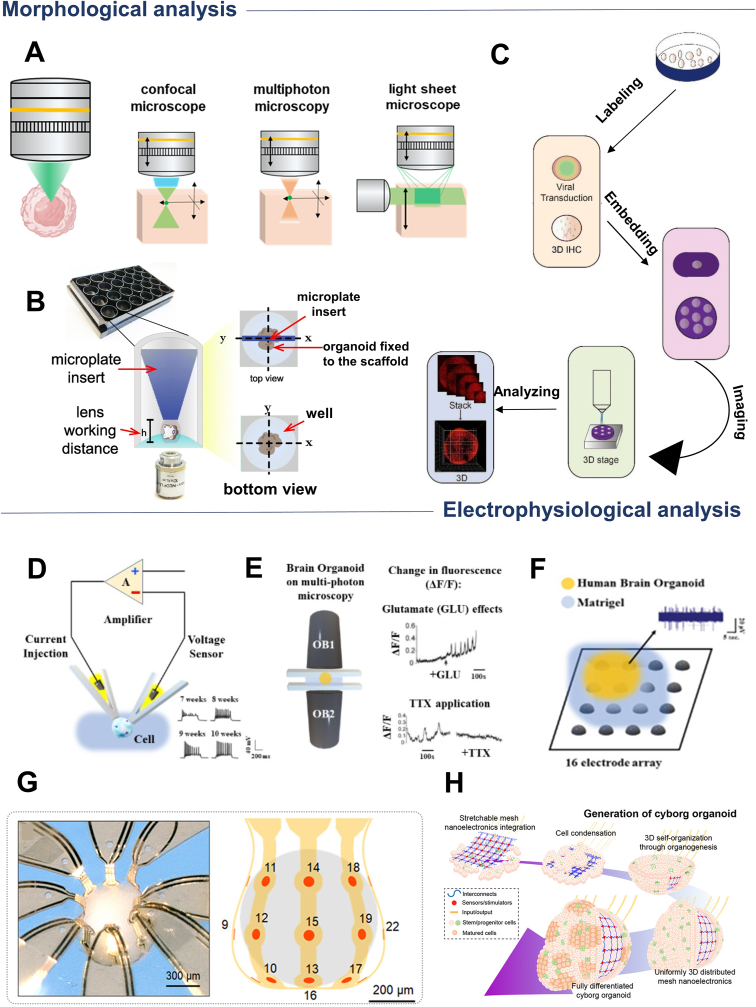
Schematic diagram of techniques used for brain organoid analysis. (A) Three commonly used microscopic techniques for morphological analysis of brain organoids: confocal microscopy, multiphoton microscopy, and light-sheet microscopy. (B) A 3D-printed microplate insert facilitates prolonged, continuous visualization of whole brain organoids. Reproduced with permission [146]. Copyright 2021, Springer Nature. (C) This is a schematic diagram of a high-resolution imaging pipeline designed for fluorescence microscopy. The pipeline facilitates optical chromatography imaging of viral transduction, fluorescent labeling, and 3D immunostaining. Reproduced with permission [134]. Copyright 2022, Biology. (D) Membrane clamp technique. (E) Calcium imaging technique. (F) Microelectrode array. Reproduced with permission [147]. Copyright 2019, Frontiers in Neuroscience. (G) 3D flexible neural interface platform for minimally invasive detection of electrophysiological properties of cortical spheroids. Reproduced with permission [139]. Copyright 2021, American Association for the Advancement of Science. (H) Integration of stretchable mesh nanoelectronic elements during the formation of brain organoids (so-called semi-mechanical human organs) for measuring electrical activity and growth throughout development. Reproduced with permission [140]. Copyright 2019, American Chemical Society.

In brain organoid cultures, the easiest way to determine the developmental stage of a brain organoid is to analyze it morphologically. However, the most intuitive morphological analysis is to utilize imaging techniques. Therefore, high-quality imaging techniques become necessary to reliably analyze the 3D brain. Confocal microscopy is the prevailing imaging device for capturing images of brain organoids, as it allows for the acquisition of fluorescent images that display the expression of immunomarkers (Fig. 7A). Confocal microscopy, though widely used for brain organoid imaging, suffers from a shallow penetration depth and absorption. As a result, brain organoids must be sliced into thin sections for millimeter-scale imaging [128], which severely disrupts their intricate 3D structure. To address this challenge, cutting-edge imaging techniques have been developed, enabling non-invasive, 3D imaging of brain organoids without the need for destructive slicing.

Because of its greater depth of penetration, multiphoton microimaging has been commonly used in recent years to study the neural structure and function of living animals, especially for imaging brain organoids (Fig. 7A). Multiphoton microscopy uses longer wavelengths, which generally results in less light scattering by the tissue. Thus, multiphoton microscopy using longer wavelengths has a greater depth of penetration than single-photon microscopy, allowing images to be captured at greater depths and reducing the risk of photobleaching and phototoxicity. For example, Rakotoson and their team developed a specialized two-photon rotating disc microscope designed with a broad field of view to enhance organoid imaging. This microscope not only operates faster but also offers deeper penetration than traditional systems, enhancing the imaging process for organoids [148]. In addition, light-sheet microscopy (LSM) is gaining popularity as a method of obtaining high-contrast, high temporal and spatial resolution images, and the imaging principle of this device is to use laser light-sheet scanning to image the sample (Fig. 7A). Since the LSM illuminates only the portion of the sample being viewed, the background signal of the biological sample is reduced, resulting in higher contrast images. In addition, LSM collects code from the light sheet simultaneously, resulting in images with higher spatial and temporal resolution. Despite advances in microscopy technology, light scattering remains a major challenge, affecting the depth of penetration and resolution of imaging.

Combining cutting-edge imaging and optical clearance techniques to develop platforms for long-term, real-time imaging of entire brain organoids would help address this problem [149]. For example, Karzbrun et al. encapsulated brain organoids in 150 μm-high microfabrication chambers on a chip and imaged the organoids over a period of weeks to observe the physical properties of brain folding [138]. The strength of this technique is its capability to enable long-term imaging *in situ*. Similarly, Mansilla and their team engineered microplate inserts using 3D printing technology, enabling the real-time, high-resolution observation of various organisms cultured within sealed compartments (Fig. 7B) [150]. The insert enables real-time tracking of the organoid and immobilization of the organoid in culture without impeding its growth.

Additionally, leveraging the strengths of *in vivo*, long-term, label-free, and 3D imaging, optical coherence tomography (OCT) has emerged as a prominent area of focus in organoid imaging research over recent years. Ma et al. designed a fluorescence micro-optical tomography technique (fMOST) capable of fluorescently labeling viral transduction and 3D immunostaining for high-resolution imaging (Fig. 7C) [134]. This pipeline is capable of acquiring millimeter-scale high-resolution organoid spatial information to analyze their cellular composition and morphology. In recent years, imaging techniques for examining the function of brain organoids have continued to evolve, ranging from common optical microscopy to advance *in vivo*, long-term and labelling-free 3D imaging methods. These developments have enabled us to observe and analyze the microstructure and cellular organization dynamics of brain organoids in greater depth in an *in vitro* culture setting and have laid a solid foundation for advancing the field toward bionic intelligence.

### 5.2 Electrophysiological analytical methods

Establishing stable neural networks in brain organoids is essential for the study of neurological diseases [136]. In order to evaluate whether organoid culture systems produce mature neurons and functional neural networks, it is necessary to evaluate their electrophysiological function during development. Given that the development and maturation of brain organoids is a time-consuming process, obtaining long-term, stable, high temporal resolution and high spatial resolution electrophysiological recordings during neurogenesis requires the development of more sophisticated measurement techniques.

Brain organoid electrophysiological analysis techniques include membrane clamp, calcium imaging, microelectrode arrays, and emerging flexible nanoelectrodes. Membrane-clamp classical electrophysiology methods enable researchers to record the individual neuron activity in brain organoids with high temporal resolution. This approach provides a detailed analysis of specific neurons, allowing for in-depth study of their function and behavior (Fig. 7D) [10,31,151]. The ability to assess responses to perturbations, such as drug treatments or optogenetic stimuli, is significantly enhanced by high temporal resolution. However, since this method primarily allows the analysis of individual neurons, it provides limited information about network connectivity or dynamics. These aspects are crucial for a comprehensive regional or overall analysis of organoid function, and therefore, complementary techniques that capture a wider range of neural network interactions are needed. Calcium imaging has been widely used to improve spatial resolution and analyze network activity. This technique allows for observing changes in calcium ions within cells that can respond to neural activity, thus allowing researchers to observe how neurons interact in networks over time (Fig. 7E). [136]. However, as a trade-off, calcium imaging sacrifices the high temporal resolution that other techniques, such as membrane clamp, can provide. In addition, calcium imaging has limitations in analyzing the 3D properties of organoids. This is because calcium imaging requires that neurons can be tightly connected in z-dimensions to capture and analyze them. This is fine for analyzing small specific areas but does not allow for global analysis.

Microelectrode arrays (MEAs) merge the temporal precision of membrane-clamp techniques with the spatial resolution of calcium imaging and are therefore increasingly used for analytical studies of brain organoids. Simultaneously analyzing extracellular potentials from a large-scale electrode array enables real-time evaluation of various network connectivity parameters (Fig. 7F). The throughput of MEA is significantly higher compared to calcium imaging and membrane clamp. Wulansari et al. used a customized microdrive system with 16 silicon neural probes integrated with microelectrodes to penetrate the internal regions of brain organoids and measure the DNAJC6 mutation associated with Parkinson’s Disease. Their analysis of neuronal activity in human midbrain organoids associated with the disease confirmed an increase in intrinsic neuronal firing frequency, aligning with the physiological characteristics of progressive Parkinson’s disease [137]. In addition, the advancement in complementary metal-oxide-semiconductor (CMOS)-based microelectrode array (MEA) technology has made it possible to record high-resolution extracellular field potentials from individual neurons across thousands of sites simultaneously at a network scale. This technique not only maintains an excellent signal-to-noise ratio, but also significantly improves the reliability of the analytical results [152].

Although 3D MEAs are valuable tools for measuring organoids, inserting conventional rigid metal electrodes can potentially damage the organoids and their neural networks. To not break the brain organoids' structure and to achieve long-term stable detection, researchers have explored strategies to interface with brain organoids electrically without electrode insertion. For example, Park and colleagues pioneered a flexible 3D neural interface platform that adapts to the contours of cortical spheres, enabling seamless integration with their structure (Fig. 7G) [139]. This design uses serpentine, deformable, and stretchable gold wires that wrap around the organoids, allowing for continuous monitoring of neural activity throughout development, regardless of the organoid’s size.

In addition, electrodes embedded in stretchable grids have become a new means of analyzing organoids by virtue of their unique advantages. These electrodes can bind to the cellular monolayer in the early stages of the organoid and then expand as the organoid matures into a 3D structure that conforms to the shape of the entire organoid (Fig. 7H) [140]. This approach has the advantage that the electrodes will be evenly distributed throughout the structure after organoid development is complete. Because the embedded nanoelectrodes do not significantly interfere with neuronal activity or cell differentiation within the organoids, this approach allows researchers to monitor the maturation process of the organoids over time. Although flexible stretchable electrodes have great potential for monitoring organoids, they still face challenges in terms of material properties and other aspects. With the rapid development of electrophysiological analysis of brain organoids, it will provide a more intelligent and effective method for the study of long-term longitudinal neural signal networks and their electrophysiological activities, and is expected to stimulate more scientific breakthroughs and innovations in the field of neuroscience.

### 5.3 Electrochemical analytical methods

The brain uses small molecules, such as neurotransmitters and neuromodulators, to transmit signals and coordinate brain functions. Therefore, detecting the release of small and medium-sized molecules in brain organoids by electrochemical method can indirectly reflect the maturity and related functions of brain organoids.

**Fig. 8:**
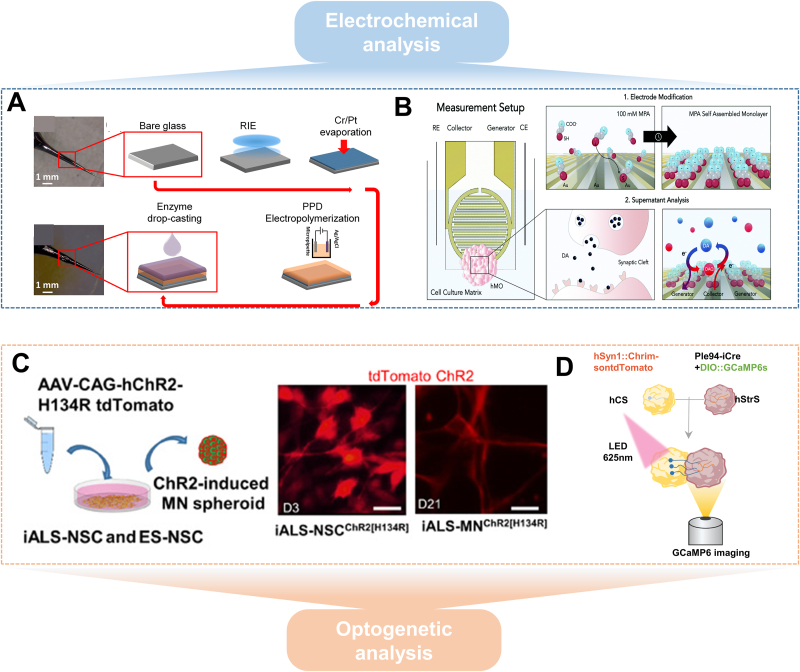
Electrochemical and genetic analysis methods for brain organoid analysis. (A) Nanostructured electrochemical biosensor for detection of neurotransmitters released from brain organoids. Reproduced with permission [141]. Copyright 2018, MDPI. (B) Redox cycle-based microsensor to monitor biomarker release from brain organoids. Reproduced with permission [154]. Copyright 2021, The Royal Society of Chemistry. (C) Optogenetics technology enabled precise spatiotemporal regulation of neural activity and muscle contractions by transfecting the light-sensitive ion channel gene into iALS-MNs and ES-derived cells. Reproduced with permission [155]. Copyright 2018, Science Advances. (D) Combination of optogenetics in calcium imaging for imaging GCaMP6-expressing neurons in the striatal globus pallidus.

The most commonly used electrochemical analytical method is the electrochemical sensor. It is very suitable for analyzing and determining small molecules in brain organoids due to its advantages of high sensitivity, good selectivity, high spatial and temporal resolution, and easy miniaturization of the detection electrode. For example, Nasr et al. engineered nanostructured borosilicate glass capillaries to create advanced electrochemical biosensors, specifically designed to detect glutamate release in brain organoids generated from hESCs (Fig. 8A) [141]. To enhance the sensors' performance, Lee et al. doped molecularly imprinted polymers (MIPs) with different concentrations and types of transition metal disulfides (TMDs) to improve electrical conductivity [153]. Zanetti et al. used a noninvasive, labeling-free electrochemical sensor to accurately detect dopamine (DA) levels in human midbrain organoids (Fig. 8B) [154]. This sensor combines a redox cycling approach with an enhanced 3-mercaptopropionic acid self-assembled monolayer, which improves the selectivity and sensitivity of the sensor to dopamine and minimizes substrate interference. In addition, Park et al. reported a 3D electrochemical microsensor capable of encapsulating organoids to measure oxygen concentration in the culture fluid near specific areas of interest [142]. This 3D architecture provides spatial mapping not available with conventional 2D electrodes and is critical for designing future brain organoid sensors. However, it is worth noting that electrochemical sensors still face challenges in terms of specificity, anti-interference ability, biocompatibility, and so on. With the continuous optimization of sensor technology, intelligent data analysis methods, and standardized processes, electrochemical analysis technology will play an increasingly important role in fields such as neuroscience and personalized medicine.

### 5.4 Optogenetic analysis methods

Optogenetics is an amazing technique for regulating cellular activity through light. This technique allows researchers to manipulate the activity of individual neurons to study the brain’s functional neural networks and the pathogenesis of related diseases.

Optogenetics has now been demonstrated to label neurons in brain organoids and to verify their maturation. Labeling of brain organoids neurons by retinoid expression generated action potentials in AAV-Syn1:ChR2-mCherry-infected cortical globular pallidum cortical spheroids when stimulated with 475 nm light, demonstrating that cultured cortical globular pallidum is functionally mature [156,157]. In addition, Mansour et al. used optogenetics to detect brain organoids that control the expression of retinol-2 (ChR2) channels, demonstrating that xenotransplanted organoids can form functional synaptic connections with the host (Fig. 8C) [29,155]. Blue laser light stimulation of the transplanted organoids via an optical fiber triggered neural activity in the host brain region, indicating that the neurons from the transplanted organoids successfully integrated functionally with the host’s synaptic circuits. Optogenetics can not only verify the integration of neurons with the host, but also test the generation of functional connectivity between two fused brain organoids by means of calcium indicators capable of responding to light stimulation. For example, Miura et al. generated a cortico-striatal assembly that expresses the retinoid protein responsive to red light in the cortical globus pallidus (hCS) and the calcium indicator GCaMP6 in the striatal globus pallidus (hStrS) (Fig. 8D) [143]. By using red light to stimulate the fusion region, a calcium response could be observed in the hStrS, indicating that neurons in the hStrS establish synaptic links with neurons in the cortical striatum [144].

In addition, the use of optogenetics in conjunction with brain organoids allows for the construction and analysis of a variety of neurological disease models to help elucidate disease pathogenesis and screen drug candidates. Osaki et al. created a 3D model of ALS using ChR2-transfected iPSCs to generate motor neuron (MN) spheroids that were co-cultivated with skeletal muscle fibers in a microfluidic device, exploring its pathogenesis and screening drug candidates through light stimulation of neuronal spheroids to induce contraction of skeletal muscles [145]. These findings suggest that the combination of optogenetics and organoids can be used to accurately study human brain organoid tissues, thereby targeting specific neuronal activities, and to establish a high temporal and spatial resolution methodology for studying cellular neural activities in order to improve the accuracy of functional analyses of brain organoids.

## 6 Diverse applications of brain organoids

**Fig. 9:**
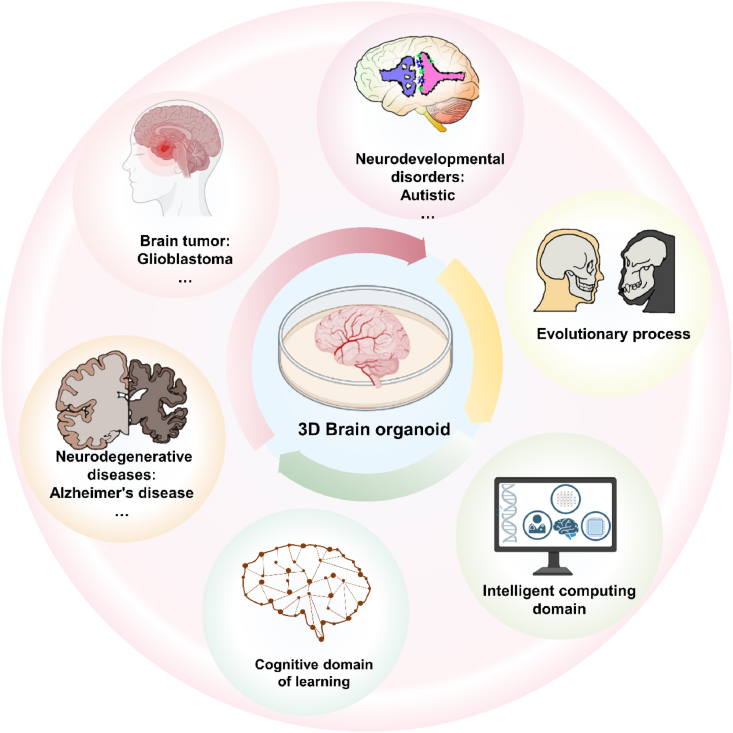
Brain organoid applications. The green arrows represent brain diseases and personalized medicine, the yellow arrows represent applying brain organoids in human evolution, and the pink arrows represent the development of brain organoid intelligence in the field of learning cognition and intelligent computing. Created with BioRender.com.

The rapid development of brain organoids has promoted their extensive application potential. This section summarizes the applications of brain organoids in the fields of brain disease modeling and personalized medicine, human evolution, and organoid intelligence (Fig. 9).

### 6.1 Disease modeling and personalized medicine

Brain organoids are used in the medical field to create disease models and provide personalized medicine based on the patient’s own characteristics [158,159]. While animal models are still the primary tool for studying human-related diseases, they cannot naturally replicate human pathologies. The emergence of *in vitro* human-specific models, including brain organoids, presents a significant advancement. These models have the potential to bridge interspecies gaps, reduce reliance on animal testing, and accelerate drug screening. The development of brain organoids provides a good scheme for *in vitro* disease research. It allows patient-specific organoids to be generated from patient-derived cells as starting material for organoid disease modeling, which will mean that organoids can be used to create personalized models of disease, contributing to the understanding of polygenic disease mechanisms, as well as providing a technological platform for *in vitro* testing of drug efficacy in patients and the development of effective therapeutic strategies [160].

For most neurological disorders, including neurodevelopmental disorders, neurodegenerative diseases, and brain tumors, it is possible to personalize treatment by building organoid models based on the patient’s own background. Autism spectrum disorder (ASD), a brain developmental disorder, presents a particular challenge, as most cases lack a clear etiological or genetic basis. This makes it difficult to replicate human brain development and impedes a deeper understanding of ASD pathophysiology. Mariani and his colleagues used iPSCs from autistic patients with megalencephaly phenotypes to generate telencephalic organoid, enabling the study of neurodevelopmental changes in patients with severe idiopathic autism. It discovered that organoids derived from individuals exhibited an accelerated cell cycle and an overproduction of inhibitory neurons [161]. The complex, multifactorial nature and pathophysiological variability of ASD pose significant challenges for clinical studies. However, iPSC-derived brain organoids from ASD cohorts provide an invaluable platform, allowing for the amplification and analysis of both cell-autonomous and non-cell-autonomous effects in a human-relevant model, offering unprecedented insights into the disorder [19,83,162]. In addition, organoids generated from homozygous lines through gene editing of specific ASD risk-associated genes offer insights into the role of individual genes within the complex genetic framework of ASD, using a single-gene approach [163].

Alzheimer’s disease (AD), a common neurodegenerative disorder, is particularly challenging to model using normal stem cells to culture brain organoids due to its late onset and the limited maturity that can be achieved in the organoids. Raja et al. proposed to construct brain organoids using pluripotent stem cells derived from the patient’s own source to mimic the pathologic features of AD, and surprisingly, it was found that in the generated brain organoids amyloid aggregation, hyperphosphorylated tau protein and endosomal abnormalities were detected [164]. This was consistent with the pathology of AD. In addition, organoids produced from induced pluripotent stem cells of individuals with familial Alzheimer’s disease inherently display significant pathological traits over the course of their development, encompassing the formation of amyloid plaques and neurofibrillary tangles. By applying β-secretase and γ-secretase inhibitors to patient-derived organoids, amyloid, and tau protein pathology is significantly diminished [165]. These findings suggest that brain organoids can recreate a complicated extracellular environment that mirrors the protein aggregate characteristic of AD patients' brains, potentially enhancing the effectiveness of preclinical drug discovery for AD [166].

Brain organoids can also recapitulate brain tumor phenotypes and complex tumor microenvironments, supporting detailed studies of the mechanisms of tumorigenesis and progression. Jacob et al. generated glioblastoma organoids (GBOs) using patient-derived cells, which recapitulate parental tumor-associated features well [167]. The organoid has the advantage of rapid generation and high reliability compared to other existing methods. When transplanted into adult rodent brains, they exhibit rapid, aggressive infiltration [168]. Moreover, linking mutation profiles with drug responses underscores the power of GBOs in advancing personalized therapies, effectively simulating T-cell immunotherapy.

In addition, brain organoids are highly effective in detecting neurophilia in viral infections. Multiple studies have shown that cortical progenitor cells are extremely sensitive to Zika virus, causing increased cell mortality and significant reduction in cortical organoid volume after infection, reflecting the clinical microcephaly phenotype [116,156,169]. Researchers have established a powerful drug screening platform by leveraging stem cell-derived neural cells to identify therapeutic agents that effectively block Zika virus infection [[170], [171], [172]]. More recently, research into the brain’s susceptibility to the SARS-CoV-2 virus has intensified due to neurological COVID-19 consequences, and organoid studies have shown the virus targets specific cell types [[173], [174], [175], [176]]. Human organoid models have become increasingly valuable for understanding specific neural cell types' susceptibility to viral infection and screening potential therapeutic agents.

### 6.2 Understanding human evolution

Human evolution has been marked by a surge in cranial capacity and cognitive intricacy, along with a substantial enhancement in cognitive abilities. The human brain can be viewed as an enlarged primate brain, approximately three times the size of that of our closest relative, the chimpanzee [177]. Therefore, understanding the primate evolutionary landscape is crucial for investigating brain characteristics unique to humans. Given the limitations of obtaining fetal brain samples, cultured brain organoids provide a promising research platform to study the development and evolution of the human brain.

*In vitro* brain organoid modeling using human and other primate iPSCs can elucidate human-specific cortical expansion that results from increased neuronal cell production [178]. Compared to macaques and non-primate mammals, human neurogenesis is extended, allowing for the prolonged expansion of proliferating progenitor cells and the accumulation of neurons, including those in the late-born supragranular cortical layers. Utilizing directed differentiation of PSCs in a 3D culture system of organoids, Otani et al. compared the generation of cerebral cortical progenitor cells in humans and three nonhuman primate species and revealed significant differences in neural progenitor output over time through clonal analysis, which revealed that human progenitor cells expand about twice as long as rhesus monkeys, and that this expansion extends to the deep neural progenitor stage, accompanied by a significant increase in neurogenetic potential and an increase in cloning size [3]. Mora-Bermudez et al. found that human subependymal region (VZ) progenitors have a longer proliferation period compared to chimpanzees, which may be an indicator of enhanced proliferative ability, further supporting evidence of increased neural proliferation in humans [179].

In addition, in combination with transcriptomics and genetic engineering techniques, organoids demonstrate effectiveness and flexibility as models for evolutionary studies. Transcriptome analysis allows us to identify developmental modules that are conserved in primate evolution. By using single-cell sequencing analysis, researchers found that expression in the human brain was also upregulated in organoids, thereby validating organoids as a model for evolutionary studies [180]. The integration of organoids and genetic engineering has shown remarkable flexibility, such that primate organoids can be “humanized” by introducing unique human genes. Based on this, Fischer and colleagues doubled the level of basal progenitor cells that play a key role in neocortical expansion, by introducing human ARHGAP11B into chimpanzee brain organoids. Furthermore, ARHGAP11B rescue experiments indicate that the lack of ARHGAP11B reduces the basal progenitor cell abundance and hinders cortical expansion [181]. Accordingly, brain organoids serve as valuable tools for studying brain evolution across species and evaluating the function of genes that have unique sequences or expression profiles in humans.

### 6.3 Organoid intelligence

Organoid intelligence combines emerging organoid technologies with artificial intelligence, ushering in a new era and allowing the use of means to accelerate cross-disciplinary discovery and insights. Artificial intelligence algorithms possess the ability to analyze the complex behaviors of organoids and dynamic stimuli responses, and this integration transforms our understanding of brains into medical strategies [182]. Additionally, it propels the advancement of biocomputing. Organoid intelligence holds the potential to revolutionize *in vitro* modeling. This advancement paves the way to make complex systems become pivotal in scientific and medical breakthroughs in the future.

Organoid intelligence is inspired by the extraordinary information-processing capacity of the human brain. This has spurred initiatives to integrate biology with computers, aiming to simulate brain function and enhance computational performance. In fact, the idea of controlling simple robots or measuring simple learning tasks through brain cell cultures has been around for more than 20 years. Shahaf and Marom, for example, reported that cultures of neurons in the primary cortex of the rat can learn because they show the expected preemptive response to low-frequency localized stimuli. Immediately after the learning curve, different electrophysiological patterns follow the stimulus. In addition, researchers have created memory and learning systems in petri dishes [183]. The findings showed that, when placed in a simulated game environment, the neurons were able to learn and demonstrate perceptual abilities. However, it is important to note that behaviors such as learning perception, while they can be called intelligence, are not the same as artificial intelligence. Artificial intelligence refers to intelligent systems created by humans that are capable of performing tasks, learning, and adapting to their environment, a process that involves non-biological systems. Computers, for example, use high-quality data sets and defined outcomes to mimic brain function.

Recently, hybrid computing systems centered on brain organoids have demonstrated the deep integration of organoids and artificial intelligence. The latest research connects brain organoids with high-density multi-electrode arrays to construct a biological *in vivo* computing platform called ‘Brainoware’ [184]. In this system, the brain organoids act not only as a computational unit but also as a dynamic physical storage pool, capable of projecting external electrical stimulation signals into a high-dimensional neural activity space, decoding these neural activity features in the output layer, and predicting and classifying them based on the raw input data. More importantly, the brain organoids possess synaptic plasticity, which enables them to adjust their functional connectivity through the input of specific electrical stimuli to achieve unsupervised learning. In addition, in practical applications, the system shows better speech recognition and nonlinear equation prediction. Compared to 2D neural cultures and neuromorphic microarrays [185], brain organoids can provide complexity, connectivity, neuroplasticity, and neurogenesis, as well as low energy consumption and fast learning for organoid biological neural networks. These unique properties of brain organoids give the Brainoware system great potential in the field of intelligent computing and open up new avenues for future biocomputing research. In the future, hybrid organic-inorganic systems may surpass current AI performance and address the shortcomings of traditional silicon-based hardware [[186], [187], [188]]. These systems could provide improved decision-making capabilities, continuous learning in tasks, as well as increased efficiency in energy usage and data processing, and show great potential for computational neuroscience, but attention should also be paid to the possible ethical issues in this development.

## 7 Challenges

**Fig. 10:**
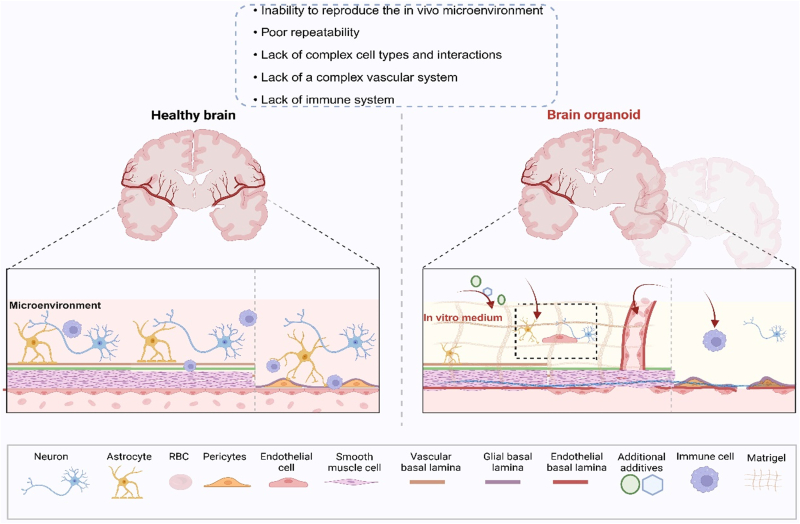
Comparison of cultured brain organoids with normal human brain. The figure shows the lack of a microenvironment in which brain organoids grow *in vivo*, the characteristic cell types, the mature vasculature, and the immune system. The inconsistency of the production process for *in vitro* cultured brain organoids limits its widespread use and leads to its low reproducibility. Created with BioRender.com.

Significant advances have been made in brain organoid technology over the past decade. Although these brain organoids can faithfully reproduce some key brain features, they are not exact replicas. Addressing the current limitations in organoid design could significantly enhance our study ability of brain development. The main problems with current organoids are as follows (Fig. 10).

(1) Brain organoids are cultured *in vitro* under limited conditions. Since most of the current culture protocols are based on previous empirical experiments or revised based on rodent brain generation protocols, they are artificial in nature and thus may omit or overuse certain factors during the culture process. It is impossible to truly mimic the *in vivo* environment of the embryo, which makes the culture conditions of *in vitro* organoids far from the *in vivo* microenvironment. Therefore, using such non-optimized models may lead to biases in our understanding and interpretation of *in vivo* neurodevelopment and the pathogenesis of certain diseases.
(2) Brain organoid production faces the problem of not being able to control the size to improve the reproducibility of the model [19,189]. Brain organoids are susceptible to the “batch effect”, in which organoids from different batches and pluripotent stem cell sources differ in differentiation efficiency, morphology, and cellular composition variability [162,190]. The heterogeneity of self-organized brain organoids is even higher, as they are completely dependent on the division and differentiation ability of the stem cells themselves. This greatly limits brain organoid modeling studies' stability and widespread application.
(3) The brain organoid cannot fully simulate the complex cell types and interactions in the body, so the brain organoid model is relatively “simple” compared to the human brain. The human brain contains billions of cells, including neurons and a variety of glial cells [191]. Although existing culture protocols are capable of producing glial cells, they do not perfectly simulate the complex interactions between cells. Especially for region-specific organoids that contain only one or a few cell types, there is a big gap between them and the human brain in terms of cell types and their spatial organization.
(4) Brain organoids do not have functional vascular networks [162]. Brain organoids can only develop properly if they are adequately supplied with nutrients and oxygenation. As a result of the restrictions posed by current cultivation protocols, the brain organoids cannot differentiate into endothelial cells to form a vascular system. The absence of a well-developed vascular system allows necrosis to occur in the center of brain organoids due to insufficient oxygen and nutrient supply, greatly limiting the size and maturation of organoids. Therefore, new techniques need to be developed to solve the problem of nutrient supply in long-term organoid culture.
(5) Brain organoid models lack the immune system [192]. An intact immune system is essential for accurately modeling conditions involving neuroinflammation and infection. However, many existing studies on brain organoid disease models do not account for immune responses. Therefore, it is necessary to incorporate the immune system into these models to better simulate the brain’s response to disease factors and pathogens.

## 8 Prospects

**Fig. 11:**
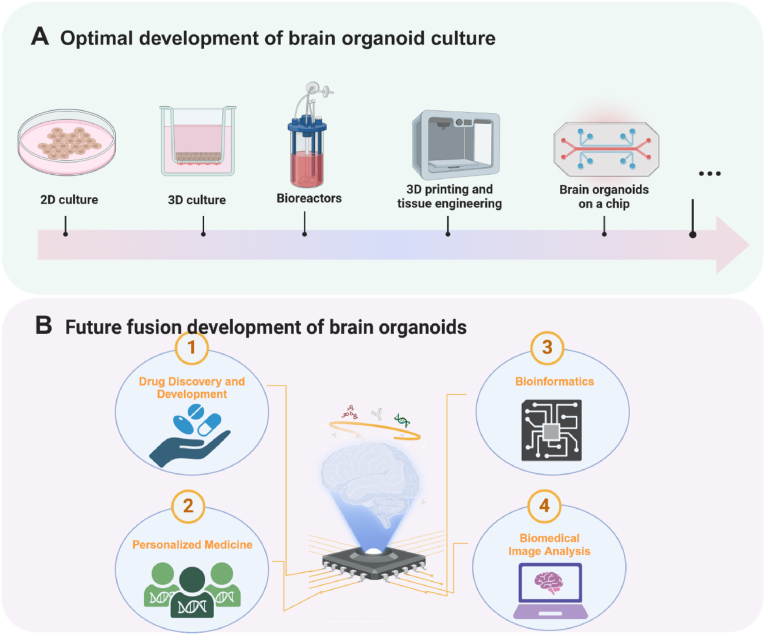
Development prospects of brain organoids. A) To optimize the development strategy of brain organoid technology, gradually shift from 2D culture to a 3D culture platform with high reproducibility, high throughput, and long-term stability. B) The future development of brain organoids combined with artificial intelligence in drug research and development, personalized medicine, and intelligent analysis will greatly accelerate brain organoids' rational design and guidance. Created with BioRender.com.

Brain organoids show a broad development prospect in the construction of human brain models, disease research, and other fields, but due to the limitations mentioned above, organoid technology faces certain limitations. In response to the problems in the above section, researchers have been exploring optimization strategies and bottlenecks in developing organoid technology (Fig. 11A). For example, to address the biomimetic problems of organoid vascularization and microenvironment, researchers have combined organoid technology with organoid chip technology, which is capable of simulating the microenvironment of human vasculature, tissues, and organs through the chip pipeline and also is capable of co-cultivating multiple organoids through modularization design, which makes it more convenient to establish the functional coupling between different organoids. In addition, 3D printing technology, microfluidic technology and other micro-nano-processing technologies are also being deeply integrated with organoid technology, which will also promote the further improvement of organoid technology bionic level in the future. Overall, the brain organoid culture system combines advanced materials, synthetic biology, and micro-nano-processing technologies to generate high-quality brain organoids with higher reproducibility, higher throughput, and feasibility of long-term 3D culture, which offers a valuable platform for the study of brain genesis, and advances the development of brain organoid modeling.

In addition, introducing artificial intelligence into brain organoid research can help improve the objectivity, accuracy, and speed of research, thus accelerating and speeding up the rational design and guidance of brain organoids. Artificial intelligence can not only provide accurate data analysis in brain organoids but also provide strong support for process monitoring and outcome prediction in drug screening (Fig. 11B). For example, through high-content microscopy images and deep learning models, researchers have been able to monitor the response of class organoids to drugs in real time, realizing non-invasive detection of drug effects and making drug screening more accurate and efficient. Therefore, brain organoid technology requires interdisciplinary research and cooperation to jointly address the challenges and promote organoid research to deeper applications. Looking ahead, organoids are anticipated to play a greater role in synergistic research in various fields, accelerating the process of their translation to the clinic and the application of precision therapy.

## 9 Conclusion

The emergence of brain organoids heralds a new era in modeling brain biology and neurological diseases, providing a powerful new platform for studying human brain specificity. However, it still has drawbacks such as heterogeneity, simplicity, and availability of long-term culture, which limits its widespread use in production. Therefore, in this review, methods and technologies to enhance the bionic function of brain organoids were reviewed from the aspects of materials biology and micro/nano processing technology, including synthetic scaffolds, microfluidics, bioprinting, and bioreactors. In addition, morphology, physiology, biochemistry, and optogenetics analysis methods are reviewed to detect the function of brain organoids more accurately, clearly, and conveniently. 3D brain organoids will become more bionic as culture techniques become more precise. Comparative studies of brain organoids with other primate brains enable a better comprehension of the evolution of the brain. Brain organoids show great potential in the fields of disease modeling, personalized medicine, and emerging organoid intelligent computing. This article provides the reader with a comprehensive overview of the development of brain organoids and suggests practical solutions to current challenges as well as possible directions for development. Particular attention is paid to the development opportunities in the emerging field of organoid intelligence. The aim is to provide new insights and considerations for the improvement of brain organoid bionic performance and the development of new fields of intelligence.

Looking to the future, by integrating brain organoids with additional engineering approaches, major breakthroughs are expected in basic sciences, including human evolution in biogenetics, computational models in mathematics, and computer science. The synergistic development of brain organoids with artificial intelligence and brain-computer interfaces in multiple fields is expected to realize the opening of new possibilities for bionic artificial robots in education, elderly companionship, medical assistance, and other fields that particularly require emotional communication.

## CRediT authorship contribution statement

**Yuli Zhao:** Writing – review & editing, Writing – original draft, Methodology, Investigation. **Ting Wang:** Methodology, Investigation. **Jiajun Liu:** Methodology, Investigation. **Ze Wang:** Writing – review & editing, Supervision. **Yuan Lu:** Writing – review & editing, Supervision, Funding acquisition, Conceptualization.

## Ethics approval and consent to participate

This manuscript is a literature review work, and thus no *in vivo* evaluations on animal model or clinical trials were performed in this scope. Thereby, our work does not fall into the incidence of ethical approvals and patient consents.

## Declaration of competing interest

The authors declare that they have no known competing financial interests or personal relationships that could have appeared to influence the work reported in this paper.
